# Persistence of Botulinum Neurotoxin A Subtypes 1-5 in Primary Rat Spinal Cord Cells

**DOI:** 10.1371/journal.pone.0090252

**Published:** 2014-02-27

**Authors:** Regina Clare Meyer Whitemarsh, William Howard Tepp, Eric Arthur Johnson, Sabine Pellett

**Affiliations:** Department of Bacteriology, University of Wisconsin – Madison, Madison, Wisconsin, United States of America; Institute Pasteur, France

## Abstract

Botulinum neurotoxins (BoNTs) are the most poisonous substances known and cause the severe disease botulism. BoNTs have also been remarkably effective as therapeutics in treating many neuronal and neuromuscular disorders. One of the hallmarks of BoNTs, particularly serotype A, is its long persistence of 2-6 months in patients at concentrations as low as fM or pM. The mechanisms for this persistence are currently unclear. In this study we determined the persistence of the BoNT/A subtypes 1 through 5 in primary rat spinal neurons. Remarkably, the duration of intracellular enzymatic activity of BoNT/A1, /A2, /A4 and /A5 was shown to be at least 10 months. Conversely, the effects of BoNT/A3 were observed for up to ∼5 months. An intermittent dosing with BoNT/E showed intracellular activity of the shorter acting BoNT/E for 2–3 weeks, followed by reoccurrence and persistence of BoNT/A-induced SNAP-25 cleavage products.

## Introduction

Botulinum neurotoxins (BoNTs), produced by neurotoxigenic clostridia, have been recognized as the cause of botulism for over a century [1]. In the past 3–4 decades BoNTs have emerged as important therapeutics for treatment of numerous neuronal and neuromuscular conditions (reviewed in: [2], [3]). BoNTs have been categorized into seven immunologically distinct serotypes, A through G [4]. Recently, introduction of an 8^th^ serotype (H) has been suggested [5], [6]; however, further research is needed to validate this classification. In recent years, many subtypes that differ in their amino acid sequences have been demonstrated for most serotypes [7]. Among the BoNT sero- and subtypes only BoNT/A1 and to a much lesser extent BoNT/B1 are available as pharmaceutical preparations. After local injection in humans, the paralytic effects of BoNT/A1 has the longest duration of action and diminish only after 2–6 months post injection [8], but the mechanism of recovery is not well understood.

For over a decade it has been known that BoNT/A1 has a prolonged duration of action in cultured neuronal cells [9]–[11], showing only partial recovery of SNAP-25 cleavage at ∼11 weeks. Conversely, cells exposed to BoNT/E possess fully recovered SNAP-25 after ∼18 days [11]. The long-term effects of several BoNT serotypes using rat cerebellar neurons demonstrated that half-lives of inhibition (correlation of neuronal cleavage with inhibition of neurotransmitter release) followed the pattern A1 ∼C1>>B1>E1>F1; (>>31, >>25, ∼10, ∼2 and ∼0.8 days, respectively) [10]. Using voluntary running as an endpoint, mice injected locally in the hind leg with BoNT/A1, /B1 or /E1 recovered after >>30 days, ∼15 days and ∼12 days, respectively [12]. *In vivo* studies after local injection in rats evaluating the persistence of BoNT/A1 and /E1 by twitch tension found full recovery for BoNT/E1, but <25% recovery for BoNT/A1 after 40 days [13]. The sequential injection of BoNT/A1 then /E1 or BoNT/E1 then /A1 both resulted in recovery similar to a single injection of BoNT/A1 with only partial recovery of twitch tension after 30 days [13]. This suggests a longer persistence and duration of action of A1 than E1 in rats. Conversely, an *in vivo* human model using simultaneous injection of BoNT/A1 and /E1 into the extensor digitorum brevis (EDB) muscle resulted in faster recovery like that of BoNT/E1 with the muscular action potential fully recovered after ∼30 days compared to only partial recovery after 90 days for BoNT/A1 [14]. This led the authors to conclude that the difference in recovery after BoNT/A1 or E/treatment is not due to different longevity of the light chains (LCs) inside the affected neurons, but rather to other unidentified mechanisms. Two major hypotheses have been put forth to explain the long-lived action for BoNT/A1 [11], [15]: (1) toxin is compartmentalized and stays enzymatically active while continuously cleaving newly synthesized SNAP-25; (2) nonfunctional, BoNT/A-cleaved SNAP-25 persists within cells for a prolonged period and prevents *de novo* synthesis of full-length, functional SNAP-25.

Within the BoNT/A serotype there exist five subtypes that have been purified and compared for various biochemical and biological properties [16]–[20], but much remains to be learned about these type A subtypes, including differences in duration of action. In a previous report our laboratory described the persistence of cleaved SNAP-25 in primary rat spinal cord cells exposed to BoNT/A1 and A2 for at least 22 weeks [21]. The goal of the work presented here was to determine the long-term duration of action of the BoNT/A subtypes 1–5, and to explore the mechanisms of persistence of cleaved SNAP-25. The results indicate that, with the exception of BoNT/A3, BoNT/A subtypes (/1, /2, /4, /5) have surprisingly long-lived activities in cultured neurons compared to clinical duration, and that this longevity appears to a result of persistence of enzymatically active intracellular toxin.

## Materials and Methods

### Ethics statement

All animal experiments were approved by and conducted according to guidelines of the University of Wisconsin Animal Care and Use Committee.

### Botulinum neurotoxin

Isolated ∼150 kDa holotoxins of BoNT/A1, /A2, /A3, and /A5 were purified from *C. botulinum* strains Hall A hyper, Kyoto-F, CDC A3 (provided by Susan Maslanka and Brian Raphael, Centers for Disease Control and Prevention) and A661222 as previously described [22]–[25]. BoNT/A4 was recombinantly expressed in a nontoxigenic strain of Hall A-hyper constructed as previously described [26], and using the nucleotide sequence from strain 657Ba [18] with a pMTL80000 vector system [27]. The purified toxins were stored in glycerol at −20°C until use, and purity was confirmed by SDS-PAGE [28]. Activities of the five subtype preparations were determined using a mouse bioassay (MBA) as previously described [29]. Specific activities of the toxins were 8 pg/LD_50_ (A1), 7.9 pg/LD_50_ (A2), 17 pg/LD_50_ (A3), 8 ng/LD_50_ (A4), and 7.3 pg/LD_50_ (A5).

### Neuronal cells

Primary rat spinal cord (RSC) cells were prepared as described [30], and plated on 0.01% poly-L-ornithine (Sigma) and 8.3 µg/cm^2^ matrigel (BD Biosciences) coated 96-well plates (TPP) at a density of 75,000 cells/well. Cells were incubated at 37°C, 5% Co_2_ and matured for 18 days prior to toxin application.

### Neuronal cell-based assays

To study the duration of action of the BoNT/A subtypes, primary rat spinal cord cells were exposed to specific BoNT dilutions sufficient to achieve ∼100% SNAP-25 cleavage after 48 h exposure in a volume of 50 µL per well (A1 = 2 U, A2 = 0.2 U, A3 = 15 U, A4 = 10 U, A5 = 25 U), as determined previously [28]. BoNTs were added in 50 µL culture medium (CM) (Neurobasal supplemented with B27 and Glutamax, Invitrogen). After 48 h, all toxin was removed and cells were gently washed twice with 200 µl warm culture medium. Fresh medium was added and the plates were returned to the incubator. Half the medium was removed and replaced with fresh culture medium twice a week. After six months medium changes were conducted every other day to avoid acidification of the medium. Cells were harvested at 48 h, 2 wks, and monthly post BoNT exposure by lysis in 75 µL 1X lithium dodecyl sulfate (LDS) sample buffer (Invitrogen) (n = 4).

To determine whether enzymatically active BoNT/A subtypes were present at 9 months post initial exposure, remaining cells were exposed to sufficient BoNT/E to generate ∼100% BoNT/E-SNAP-25 cleavage (10 U/50 µl), i.e. cleavage of both uncleaved and already A-cleaved SNAP-25. Cells were washed gently to remove remaining toxin, and harvested at 48 h and in 5-day increments as described above.

Recovered cells exposed to BoNT/A3 maintained in culture for 9 months post toxin exposure and negative control cells not exposed to toxin maintained in culture for 10 months were monitored for their sensitivity to BoNT/A1. Cells were exposed to serial dilutions of BoNT/A1 in 50 µL of culture medium for 48 h (n = 4), and a negative control where toxin was omitted. Cells were lysed and harvested as described above.

Cleaved and uncleaved SNAP-25 bands were quantified by densitometry using a Foto/Analyst FX system and TotalLab Quant software (Fotodyne) [28], [31]. Data plots and best-fit lines (four parameters – variable slope) were generated using PRISM 6 software.

Statistical Analysis: One-way ANOVA with a 99% confidence interval was used to compare the rates of recovery of SNAP-25 cleavage in cells treated with BoNT/A1-5. A two-way ANOVA with a 99% confidence interval was used to determine compare the percentage of cleaved SNAP-25 before and after BoNT/E treatment after 9 months in culture.

## Results

### Recovery of SNAP-25 in neurons exposed to BoNT/A subtypes

**Figure 1:**
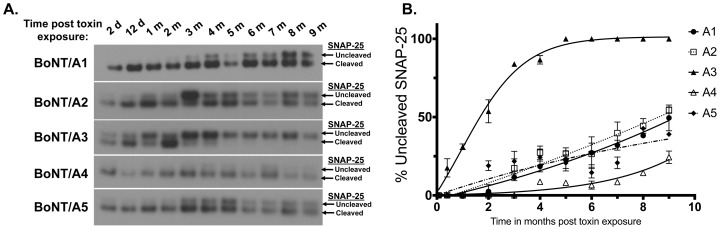
Recovery of SNAP-25 after exposure to BoNT/A subtypes. Primary rat spinal cord (RSC) cells were exposed individually to sufficient concentrations of the BoNT/A subtypes 1 through 5 to generate ∼100% cleavage of SNAP-25 after 48 h. Excess toxin was aspirated and cells were washed to remove any remaining extracellular toxin. Cells lysates were collected at the time points specified and were analyzed for SNAP-25 cleavage by Western blot, and a representative blot is shown (**A**). Data from 4 Western blots were quantified by densitometry, and data plots generated using PRISM software (**B**).

To determine whether BoNT/A3, /A4, and /A5 subtypes have similarly long durations of action as seen previously with BoNT/A1 and BoNT/A2 [9], primary rat spinal cord (RSC) cells were exposed to the minimal amount of toxin required to generate ∼100% BoNT/A-induced SNAP-25 cleavage (SNAP-25^A^) within 48 h. It has previously been determined that the sensitivity of RSC cells differs for BoNT/A1-5, and the minimal amount of each subtype to achieve 100% SNAP-25 cleavage has been determined empirically [28]. After removal of extracellular toxin by washing, and further incubation of the cells, samples of cells were harvested monthly and analyzed for cleavage of SNAP-25 over a period of 10 months. Nearly 100% cleavage of SNAP-25 was observed after 48 h exposure to BoNT/A subtypes 1–5 (Figure 1). BoNT/A3-treated cells showed the fastest and least gradual recovery of uncleaved SNAP-25 starting at 2 weeks post exposure, and contained only uncleaved SNAP-25 at ∼5 months (Figure 1). Cell exposed to BoNT/A1, /A2 and /A5 recovered very slowly and gradually over time starting at 2–3 months and reaching ∼50% uncleaved SNAP-25 after 9 months (Figure 1). BoNT/A4-treated cells showed the slowest start in recovery of SNAP-25, with only about ∼25% uncleaved SNAP-25 detected after 9 months. Once recovery started, the slopes of the curves for BoNT/A1, 2, 4, and 5 were not significantly different as determined by one-way Anova (p = 0.01), whereas the slope of the curve for A3 was significantly greater. This indicates a similar rate of recovery for BoNT/A1, 2, 4, and 5, but faster recovery for A3.

### The BoNT/A1, 2, 3, and 5 light chain activity persists in cultured neurons past 10 months

**Figure 2:**
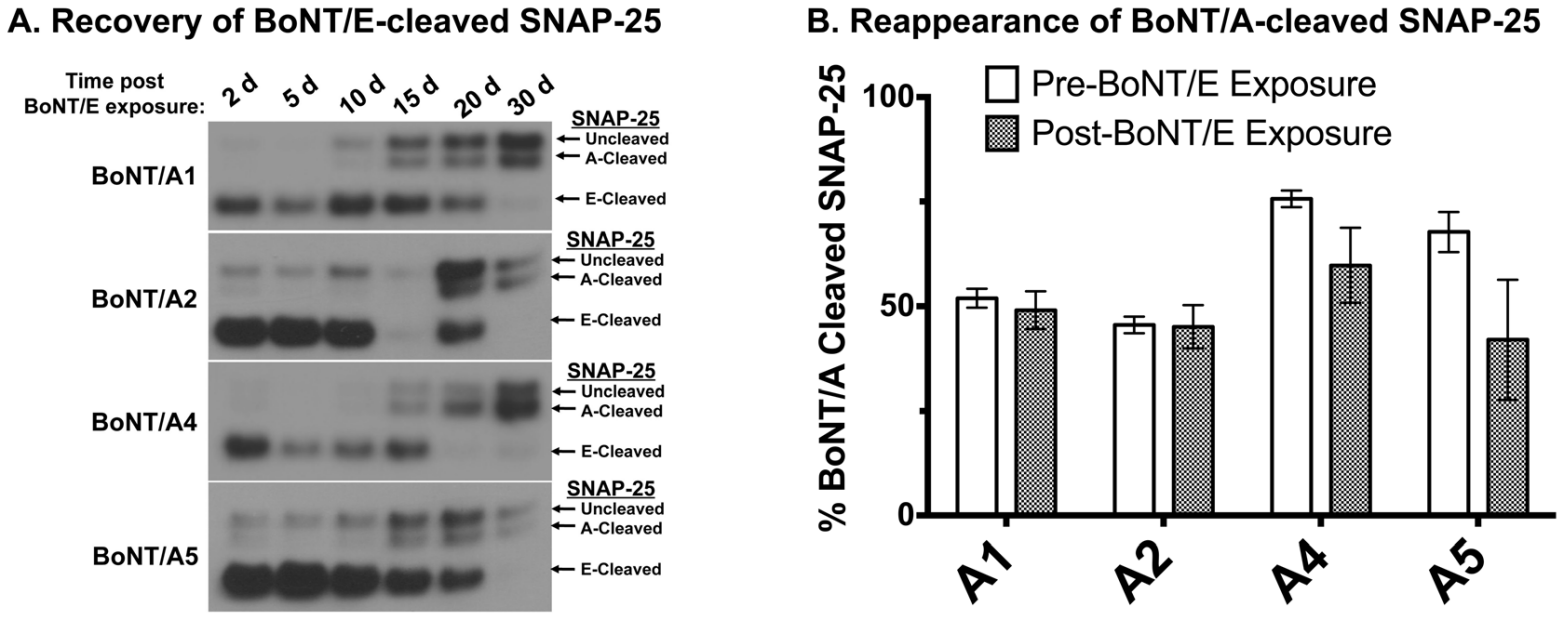
Secondary intoxication with BoNT/E to monitor persistence of BoNT/A light chain activity. Primary rat spinal cord (RSC) cells that had been exposed to BoNT/A1, /A2, /A4, and /A5 and maintained for 9 months were exposed to 10 U/50 µL of BoNT/E, a sufficient concentration of toxin to cleave ∼100% of both uncleaved SNAP-25 and SNAP-25^A^ after 48 h. Excess toxin was aspirated and cells were washed to remove any remaining extracellular toxin. Cells lysates were collected at the time points specified and analyzed for SNAP-25 cleavage by Western blot. A representative Western blot is shown (**A**). Data from 4 Western blots were quantified by densitometry, and a graph showing the percentage of SNAP-25^A^ before and 30 days after BoNT/E exposure (when no SNAP-25^E^ remained) was generated using PRISM software (**B**).

To evaluate the hypothesis that the persistence of SNAP-25^A^ was a consequence of enzymatically active light chain, partially recovered cells at 9 months post BoNT/A1, 2, 3, and 5 exposure were exposed to BoNT/E and monitored over time for reappearance of uncleaved and BoNT/A and /E-cleaved SNAP-25. BoNT/E exposure resulted in ∼100% BoNT/E-induced cleavage of SNAP-25 (SNAP-25^E^) after 48 h (Figure 2A). Over the next 30 days, the SNAP-25^E^ completely disappeared, and uncleaved SNAP-25 and SNAP-25^A^ recovered to a similar ratio as observed at 9 months (Figure 2B). The uncleaved SNAP-25 to SNAP-25^A^ ratio showed minor increases for A1, 2, and 4, and a significant increase for A5, indicating a continuing trend for slow recovery of uncleaved SNAP-25. A similar study was conducted at 4 months post BoNT/A1 and 2 h exposure with the same results (data not shown).

### RSC cells remain sensitive to BoNT/A1 after 10 months in culture

**Figure 3:**
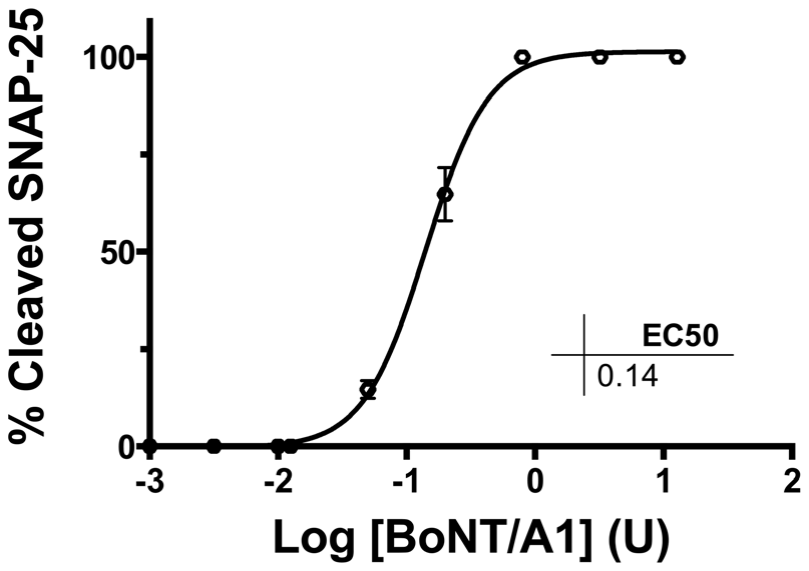
Sensitivity of RSC cells cultured for 10 months to BoNT/A1 after 48 h exposure. Cells were exposed to the indicated concentrations of BoNT/A1 and cell lysates were analyzed for SNAP-25 cleavage by Western blotting. Data from three Western blots were quantified by densitometry, and data plot and an EC_50_ were generated.

To determine if the recovered BoNT/A3-treated cells were still susceptible to BoNT/A intoxication, recovered A3-treated cells with fully recovered SNAP-25 were exposed to serial dilutions of BoNT/A1 at 9 months post initial BoNT/A3 exposure. These cells showed sensitivity equal to RSC cells matured for 2 weeks with an EC_50_ of ∼0.2 U (data not shown). Similarly, previously non-exposed cells maintained in culture for 10 months, in parallel with the BoNT/A exposed cells and from the same cell preparation, were exposed to serial dilutions of BoNT/A1 for 48 h. These cells also were similarly sensitive to BoNT/A1, with an EC_50_ of ∼0.14 U (Figure 3), indicating that cultured rat spinal cord cells do not lose sensitivity to BoNT/A1 for at least 10 months in culture.

## Discussion

The results presented here show that BoNT/A3 has significantly shorter duration of action in RSC cultured neurons than BoNT/A1, /A2, /A4, and /A5, and that the LC activities of BoNT/A1, A2, A4 and A5 are extraordinarily long-lived, persisting for over 10 months. To our knowledge, the susceptibility of cells maintained in culture for 10 months is the longest ever tested in neuronal primary cell lines.

In 1998, Eleopra *et al.* observed that BoNT/A1 injected into human EDB muscle resulted in long-lasting (over 3 months) paralysis, whereas injection of BoNT/E1 resulted in a much shorter duration of paralysis with recovery starting at 14 days [14]. Simultaneous injection of BoNT/A and /E resulted in the same duration of action as injection of only BoNT/E, leading to the hypothesis that the longer duration of action of BoNT/A is not due to persistence of the LC within the neurons [14]. This was followed by two reports published in 1999, which both posited three primary hypotheses for the observed differences of duration of action between BoNT/A (over 30 days in vivo and over 11 weeks in cultured neurons) and compared to shorter duration of BoNT/E (14 days in cultured neurons) using *in vivo* and *in vitro* approaches: (1) The BoNT/A light chain (LC) has a longer-lived enzymatic activity than BoNT/E [11], [15]; (2) the cellular repair machinery responds more quickly in response to the larger BoNT/E cleavage product (26 amino acid cleavage product for BoNT/E compared to 9 amino acids for BoNT/A), resulting in degradation of the cleaved SNAP-25 and synthesis of new full-length SNAP-25 [11], [15]; or (3) cleavage of SNAP-25 incites cellular changes to the original nerve terminal that are only overcome after a long-term remodeling process [15]. Several reports have been published since then exploring the differences between BoNT/A and /E using *in vitro* and *in vivo* approaches [13], [32]–[34].

Our study used cultured primary rat spinal cord cells (RSC cells) to determine the duration of action of subtypes BoNT/A1-5. Spinal cord neurons exposed to BoNT/A1, /A2, /A4, and /A5 showed partially recovered uncleaved SNAP-25 very slowly and gradually over the 10 months (Figure 1 and Figure 2). After 10 months, at least 50% of SNAP-25 remained cleaved, indicating a duration of action of over 10 months for these BoNT/A subtypes in cultured neurons (Figure 1). The recovery of BoNT/A-cleaved SNAP-25 30 days after BoNT/E exposure of these neurons (previously exposed to BoNT/A1, /A2, /A4, or /A5) at month 9 of the study indicated that the continued presence of cleaved SNAP-25 is due to persistence of enzymatically active LC in the neurons. Keller *et al.* previously used similar experiments to evaluate persistence of BoNT/A1 LC in cultured neurons at 2 months post exposure [11], and we have also confirmed this at 5 months post exposure for BoNT/A1 and /A2 [9]. In our experiments all detectable SNAP-25, including BoNT/A-cleaved SNAP-25, was cleaved by BoNT/E1, which cleaves 26 amino acids from the C-terminus including the BoNT/A cleavage site. The reappearance of BoNT/A cleaved SNAP-25 upon recovery could only be due to cleavage of *de novo* synthesized SNAP-25 by BoNT/A1 LC still enzymatically active within the neurons after 10 months in culture. Therefore, these data suggest that enzymatically active BoNT/A LC persists in intoxicated neurons that continuously cleaves newly synthesized SNAP-25, and that the LC is very slowly degraded or removed from the cell cytosol over time. It is not clear if the enzymatically active LC is present in all spinal cord cells throughout the study, or if there is active LC in only a subpopulation of neurons. Furthermore, study of neurite sprouting of intoxicated cells might lead to identification of new cellular compartments in which newly synthesized SNAP-25 is not accessible to BoNT/A LCs. While previous reports indicate distribution of the BoNT/A LC throughout the cellular compartments after intoxication [35], one report indicates that the LC remains localized to intoxicated nerve terminals and does not affect newly formed neurite sprouts [15].

Spinal cord neurons exposed to BoNT/A3 contained 100% uncleaved SNAP-25 at 5 months (Figure 1). The molecular mechanisms underlying the faster recovery of A3-treated cells compared to other subtypes is unknown. We hypothesize that the A3 LC, which contains the greatest amino acid dissimilarity compared to A1, has an overall reduced intracellular stability. Furthermore, our study suggests that recovery of SNAP-25 in cells exposed to BoNT/A4 started significantly later but proceeded at a similar rate than in cells exposed to BoNT/A1, /A2, or /A5. While a greater stability of the BoNT/A4 LC cannot be excluded at this time, it is most likely that the observed delay in recovery is due to the fact that those cells were exposed to an ∼1,000-fold greater molar concentration of toxin than the other subtypes to achieve 100% SNAP-25 cleavage. BoNT/A4 has an ∼1000-fold lower specific activity compared to BoNT/A1 as determined by the mouse bioassay (MBA), and also requires about 1,000-fold greater molar concentration in neuronal cell models to achieve SNAP-25 cleavage [28].

Both RSC cells previously intoxicated and fully recovered (exposed to BoNT/A3, recovered, and maintained in culture until 9 months) and negative control cells (maintained in culture for 10 months), were equally sensitive to BoNT/A1 induced cleavage of SNAP-25 with an EC_50_ of ∼0.3 U. This sensitivity is equivalent to RSC cells matured for 2 weeks consistent with previous reports [28], [31]. From these results we concluded the RSC cell population remains fully sensitive to BoNT/A, even after 10 months in culture. Additionally, the morphology of the cells changed only slightly to contain more aggregates and neurites and connected by axon-like structures (not shown), still resembling neurons after 10 months.

The persistence of enzymatic activity of BoNT/A subtypes in cultured RSC cells for over 10 months, compared to shorter durations of action in animals [12], [15] and humans [14] is interesting and requires further exploration. It is currently not known how much functional SNAP-25 is required for neurons to be able to perform exocytosis and transmit a signal, but it is hypothesized that the cleavage of even a small percentage of SNARE proteins results in inhibition of exocytosis [36], [37]. Meunier *et al*., observed that intramuscular injection in the hind limb with 5 pg of BoNT/A1 resulted in ≤12% SNAP-25 cleavage, yet a maximal loss of the toe spread reflex in mice lasting for 28 days, which was accompanied by the formation of functional nerve sprouts [37]. The *in vivo* formation of nerve sprouts in BoNT/A1-intoxicated neurons [37] may result in new SNAP-25 that is not accessible to the BoNT/A LC, if the LC remains largely localized to intoxicated nerve terminals, as previously suggested [15]. This is in agreement with previous reports indicating the distinct localization of BoNT/A LC in several cell models [35], [38]. The data presented here quantified the percentage of SNAP-25 cleavage in total cell lysates from cultured neurons, which could not distinguish between newly synthesized SNAP-25 in the original nerve terminal compared to new nerve sprouts. Additionally, in the experiments presented here nearly 100% cleavage of SNAP-25 was achieved in the RSC cells, which likely contributed to the long persistence of LC activity in the model used here. It is possible that a combination of these two mechanisms is responsible for the observed durations of action *in vivo* and in cultured cells.

The persistence of BoNTs in neuronal cells warrants detailed studies to reveal the underlying molecular mechanisms. This long-lived persistence is reminiscent of the ability of some neurotropic immune-targeting viruses, such as herpes simplex virus, human immunodeficiency virus, varicella-zoster and polio to name a few, to persist *in vivo* inside neuronal cells for many years [39]. Interestingly, BoNTs share some features with viruses in that some proteins are synthesized as polyproteins, which are then post-translationally cleaved to their active forms, leading to the speculation that BoNTs may share some evolutionary relatedness with human viruses [40], [41].

The data presented here demonstrate that long-term persistence of BoNT/A-cleaved SNAP-25 is a result of persistence of enzymatically active light chain within cells, but it remains unclear how the LC evades degradation. Previous reports have hypothesized that shorter-lived BoNT/E LC is degraded by the ubiquitinin-proteasome system while BoNT/A1 LC remains stable [42]. Additionally, examination of intracellular localization of endogenously expressed GFP-labeled BoNT/A and /E LCs showed that regardless of the cell type, A1-LC localized in a punctate manner to discrete areas of the plasma membrane while GFP-labeled E1-LC localized to the cell cytosol with nuclear exclusion [35], [38]. Based on these reports, we hypothesize that the BoNT/A1 LC may evade ubiquitination and degradation through membrane association such that the N-terminus is not accessible to ubiquitination. Alternatively, it is possible that another slower acting protein degradation pathway is involved, and that an unidentified association with proteins may promote the remarkable persistence of BoNT/A in neurons.
